# Thoughts on the Philosophy of Homœpathy and Correlated Subjects

**Published:** 1895-07

**Authors:** J. W. Thomson

**Affiliations:** Chairman of the Bureau of Homœopathic Philosophy of the Society of Homœpathicians


### Bureau of Homœopathic Philosophy.

J. W. THOMSON, M. D., CHAIRMAN.

---

### THOUGHTS ON THE PHILOSOPHY OF HOMŒOPATHY AND CORRELATED SUBJECTS.

By J. W. Thomson, M. D., Chairman of the Bureau of Homœopathic Philosophy of the Society of Homœopathicians.

[Read at the Annual Meeting, Oriental Hotel, Manhattan Beach, June, 1895.]

---

### PREFACE.

We cull from *The Genius of the Homœopathic Healing Art*, being part of the preface to the second volume of *The Materia Medica Pura* (1833), by Dr. Christian Friedrich Samuel Hahnemann, translated by Dr. Adolph Lippe, and published in *The Organon*, p. 246, *et seq.*, July, 1878, the following:

"The life of man * * * cannot be compared with a wheelwork, with a hydraulic machine, with chemical processes, with decomposition or formation of gases, with a galvanic battery, or with anything inorganic."

"Life is in *no respect* controlled by any physical laws, which govern only inorganic substances. The material substances composing the human organism are not governed in their living composition by the same laws to which inorganic substances are subjected, but they follow solely laws peculiar to their vitality; they themselves are animated and vivified, just as the whole organism is animated and vivified. Here reigns a nameless all-powerful fundamental force, which suspends all forces of the constituents of the body inclined to follow the laws of pressure, collision, depression, fermentation, and decomposition, and only this force guides and governs by the wonderful laws of life; that is to say, it maintains the necessary conditions for the preservation of the living whole in sensation and action, and that in an almost spiritual dynamic condition.

"As the organism in its normal condition depends only on the state of its vitality, it follows that the changed condition which we call sickness must likewise depend not on the operation of physical or chemical principles, but on originally changed vital sensations and actions; that is to say, a dynamically changed state of man—a changed existence—through which eventually the constituent parts of the body become altered in their character as is rendered necessary in each individual case through the changed conditions of the living organism."

This language of Hahnemann involves the most deep, profound, and far-reaching philosophy.

While keeping our feet on *terra firma* we soar into regions of which the materialist can have no conception while he remains engrossed with the husks of things.

As the stories of a house so may we say are the regions of the mind. Some are content to dwell in the basement and cellar. They have a certain kind of pleasure, with which they appear content. Could they be persuaded to open the doors and windows of these higher regions of their mental house they would begin to see broader and deeper correspondential relationships between themselves and nature than they had conceived of. Like one who had always lived in a dungeon, when their mental sight became accustomed to the brighter light and broader vision and the mold, mustiness, and earthy smell on themselves and their garments gave place to the sweetness of the purer air and clearer light, they would begin to breathe more deeply and have a desire for finer pabulum than had hitherto contented them. It would be like a new birth of mental, moral, and spiritual relationships of which they had no conception.

To this feast of fat things we invite all who love the truth for its own dear sake. 'Tis sad, indeed, to see so many groping in the gross outer darkness of mere materiality. The road at first will be very hard to travel, and they may wish themselves back to their old haunts, comforts, and flesh-pots. For a long time it will be no bed of roses; but toilsome, narrow, and difficult the path to attain that which will ultimately crown them, not with worldly advantages, but with a spirit with itself content, springing from noble uses in lieu of injury, or at best, negative results in their professional career.

About the last *ignis fatuus* they eagerly followed was the antitoxin craze. They fell over one another to get at it. A leading New York daily headed a subscription list with one thousand dollars, and the poor horse was tortured in the supposititious interest of humanity. All this would be amusing if it were not so sad. Some undoubtedly lost their lives from the injections. Many had their blood poisoned, entailing future susceptibility to disease.

Then, at a stated meeting of the New York Academy of Medicine, April 4th, 1895, one of the members, whose name we honor, took the bull by the horns, or rather showed conclusively the fallacy of the last red herring that had been drawn across their trail. And the whole business collapsed. And thus will it ever be—as it ever has been—while they attempt to treat this, that, or the other disease in detail and from apparent external causes, instead of searching for the law that is correlated to every disordered condition that can possibly affect humanity.

In support of the view that the law of cure has been discovered, and that *similia* is that law, we invite your attention.

NEW YORK, June 26th, 1895.

### THOUGHTS ON HOMŒOPATHIC PHILOSOPHY.

"Wisdom is the principal thing; therefore get wisdom: and with all thy getting get understanding.

"To investigate Nature by her own light * * * without some previous and commanding doctrine of man, connecting her with God, is like putting to sea without a compass."

There is no subject having reference to human welfare in this world, and our capacity for use to ourselves and to others of greater value and importance than that of medicine. The necessity for its use may be said to be universal. Between the cradle and the grave there are very few, if any, to whom its right administration would not be useful.

In this age of discovery and invention everything is on the move. A machine or instrument to shorten labor and increase production is brought forth, and straightway thousands are enabled to enjoy what hitherto had been the privilege of a small number. A continent is discovered or opened that gives to teeming millions opportunities for life and progress never before dreamed of. A law only vaguely thought of or desired by some patient, plodding student in the realm of mind is conceived, and though he bring not silver or gold from the storehouse of thought, ennobled by the love of use to his fellow-man, he brings what shall be of infinitely more value—the knowledge of law to improve, to elevate, and to bless humanity. Never did discoverer bring nobler gift than we here present; a gift that shall be the means of restoring order to this human organism, making it a suitable habitation for the mind; a house of order, in which and through which all the faculties—intellectual, moral, and spiritual—may obtain fruitions of development never before conceived of. This, and more than this, Hahnemann was the means of doing when he discovered Homœopathy.

But truth, mercifully, never forces itself on the human mind. We are free to accept or to reject; and we are also free to only partially accept. Between cordial acceptance and absolute denial there are many stages and degrees. We are also free to twist, gnarl, or deform the truth, as imperfect glass distorts the forms of objects which pass through it to the eye. We may also defame, even while partially using, and mix it with the errors in which we were brought up or have acquired. There are those who remain in the old camp, joining loudly in the chorus against the new, yet secretly trying to reap whatever advantages it may bring. For a long time only a few earnest, faithful souls, in love with the truth for its own sake, will accept and strive to use it in all its plenary fullness.

Probably the first thing that led to the conception of the Homœopathic law in Hahnemann's mind was in translating Professor Cullen's *Materia Medica* in 1790; that to him we will assume was the same as the fall of the apple to Newton. In 1805 he published two volumes in Latin on the power of drugs—*i. e.*, their effects on the healthy body. Then Homœopathy was born, although not christened until 1807, when he first used the word "Homœopathic" in Hufeland's *Journal.* Eighteen hundred and ten has been said to be the birth-year of Homœopathy, because that was the time of the first edition of *The Organon.* But we opine that infants do not give the most profound statement of principles, and rear a philosophy thereon at birth; therefore Homœopathy was a stalwart boy at least by this time.

Hahnemann saw that there must be a history of drug-action on the human organism that should "a round, unvarnished tale deliver" free from all physiological or pathological technicalities that might cloud or obscure phenomena; that no matter what so-called advances there might be in the kindred sciences that are the hand-maidens of the healing art, that that God-given science and art should forever stand on an impregnable foundation. Henceforth for those who desire and love the truth there was no more confusion and chaos in the therapeutist's art: it was no longer a tangled skein of doubt and uncertainty. They read no longer like—

> "An unpracticed swimmer, plunging still
> With too much labor, drowns for want of skill."

Unfortunately it is their patients who drown when the allopathic doctor will not accept and strive to live up to the light of *law* that hath come into the world.

It might be well to outline some brief definition of what we understand by natural *law*. Many definitions have been attempted. William Sharp, M. D., F. R. S., in a brochure on the *Principles of Homœopathy*, thus defines it: "It is to be understood to be the will of the Creator in the physical government of His own works." Our only objection to this is its lack of clearness, definiteness. We ask what is meant by the *will* of the Creator? People have many and diverse views of the Divine Will. Recently we heard a minister in a sermon speak of God the Father, Son, and Holy Ghost as sitting on three thrones before the creation and consulting as to how they were to proceed. Many have the idea of creation as the exercise of arbitrary power on the part of the Divine Being.

What, then, do we understand by natural law? We venture the following definition:

The orderly government of the natural universe by the Divine Being, through and by discrete degrees.

While this expresses our thought there may be some who do not reach our meaning. For greater clearness, we briefly explain. So far as the human mind can apprehend the Deity, as revealed, He is infinite Love, Wisdom, and Power; just as a man, finitely, when in order, is affection and intelligence, and from these performs the uses of his life.

> "And well it stamps the Human Race,
> And hence the gift TO UNDERSTAND,
> That man within the heart should trace
> Whate'er he fashions with the hand."

Just as the mind through the brain, the heart, and the lungs, in series and degrees, makes this human form with all its God-given powers and adaptability, we reach above and beyond the conception of mere arbitrary fiat to adequate first cause, that finds orderly correspondential expression. Thus, and not otherwise. The only, and therefore the best possible. The Infinite from His own absolute fullness and power created the universe and all that therein is, as the finite expression of Himself and man as the complement and *summum bonum*, in His own image and likeness. Seeing that creation is continual preservation, He is now creating momentaneously; as the orderly human mind is ever by mental and physical appropriations, differentiations, and rejections sustaining the body; as the natural sun is ever creating and sustaining the natural world. It may now, we think, be more clearly seen what we understand by *natural law*, which we further explicate, as:

The constant and unvarying expression of the Love, Wisdom, and Power of the Infinite, through suitably created mediums in the ultimate or lowest—*i. e.*, the natural plane of existence.

A science must be capable of infinite progress in each of its elements, without infringement of its integrity. And within its own domain it must provide for the prediction of future events. Thus, the recent discovery of Argol has not shaken our faith in the law of chemical affinity, neither could it if a dozen new elements were discovered in the atmosphere. We still predict that given certain elements and the corresponding phenomenon is inevitable.

This is beautifully expressed by the late Dr. Carroll Dunham: "Given the therapeutic law and a certain series of phenomena of natural disease to find the corresponding series of phenomena of drug disease."

Each of these may be pursued separately; the former as pathology, the latter as pathogenesy.

Now, inductively there can be no end to the progress of the elements that meet to form the science of therapeutics. All the modern progress in the knowledge of pathology has not altered and cannot alter the mutual relation which the totality of the symptoms of disease bears to drug-action. The wider, deeper, and more thorough the vista we have of pathology, the firmer becomes the basis of our synthesis for the science of *similia*, and hence, *nota bene*, ever the more must we firmly, unflinchingly adhere to the strictest individuality in our treatment, never forgetting that each patient must be studied as a diverse entity, and that the expression of each disordered state has individual peculiarities it is our province to ascertain; and our success as healers can only be assured in the measure in which with singleness of purpose we earnestly keep this thought before us.

Disease cannot be treated by nomenclature, just as you cannot reason from chemistry to gravitation. It is quite correct to give a certain array of disordered conditions a name, such as measles, diphtheria, ague, pneumonia, etc. The error begins when we make diagnostic classification also do duty therapeutically and assume that because our diagnosis is accurate, therefore we are on the way to true treatment, and have only to select the remedy or mixture available for the disease before us. The true thought is, that disease is as varied in its expression as the individual; thus, when we proceed from diagnosis to therapeutics, we enter a new field of study and must begin *de novo*, as we do when we pass from the consideration of normal to that of abnormal conditions. Each patient must be studied as an entity differing from all others, and the remedy must be in correspondence with the patient *per se*. Hence the crazes to find remedies for this, that, or the other disease by name are simply the pursuit of shadows forever eluding the grasp, and have no more foundation in reason than the chasing of a rainbow to get the bag of gold at the end of it.

Hahnemann cured seventy-two out of seventy-three typhus patients at Leipsic. And it was doubtful whether the seventy-third patient died of the disease or of old age. Dr. Adolph Lippe treated over 150 cases of scarlet fever, in 1859, at Carlisle, Pa. MORTALITY, NONE. The allopaths lost ninety per cent., and the survivors were crippled for life. (See *Cincinnati Medical Advance*, 1876, Vol. III, p. 544.)

In 1871 and 1872 Dr. Lippe also treated a large number of cases of small-pox in Philadelphia, using the higher potencies of the homœopathic remedies solely, and made no external applications whatever; did not lose a case and NOT A CASE PITTED. (*Homœopathic Times*, 1877, Vol. V, p. 187.)

Thousands of cases showing the overwhelming advantages of pure Homœopathy might be adduced, but time and the necessity for brevity forbid. We only wish to emphasize the fact that the *law* of *similia* is paramount, and the only true guide to therapeutic practice. Those who have the education, the special ability and talent, and whose minds trend toward the love of truth for its own dear sake and wish to practice it, may know whereof we speak. But there is no royal road to this or to any true knowledge. It is not sporadic, like the elixir of life, antitoxine, or the scores of things that, like Jonah's gourd, grow in a night and are short-lived as the ephemera. It demands singleness of purpose and long years of patient thought and toil to attain these blessed results; but being attained, we reach a continent of supreme use, wherein dwells the love of humanity.

There is no wonder that Hahnemann, having his car of uses tethered to the horses of his intellect by the golden chains of the love of use, should soar into the empyrean where are the higher regions of the mind, and strive to see the connection of things as they abide in their *causa causans*, the world of mind. He speaks of the light of Jupiter fading before the god of day as an illustration of the weaker, disorderly disease fading before the more powerful dynamic remedy. It has been said that this is only speculation, and that they are not parallel cases. But we know from actual experience that the remedy does drive out and disperse disorder; must it not by parity of reasoning be stronger than that which it subdued? True, it allies itself with the nerve centres to perform its mission of love; but the nerve centres are in the toils, and alone cannot pursue the fight. So the light of Jupiter, being without heat, is hidden when the greater light of the sun, burning with the loves of uses, makes its appearance.

We cannot give up one jot or tittle of the claims of God's law. When Constantine Hering said: "If our school ever gives up the strict inductive method of Hahnemann, we are lost," he stated the truth with reference to all the renegades of our school. But we will not believe that when the Lord in His own good time has sent His messenger to discover the truth, that He will fail of armor-bearers for His standard. We need have no fear for the law of *similia: in hoc signo vinces.*

Those who aim to practice the God-given law of *similia* in its integrity have been accused of looking upon Hahnemann as a sort of demi-god, and the accusers proceed to say that Homœopathy is not everything, that there are other things necessary, that Homœ-

opathy is all very well in its place. These people assume a sort of judicial *rôle*, and present the appearance of extreme wisdom and impartiality to those who cannot see through their pretenses. They make loud claims to moderation in all things, and thus by their blind adherents are looked upon as the salt of the earth. There are probably few words that have been more abused in their true meaning than this word moderation. It has been distorted, and made to apply to principle in lieu of conduct, etc. Thus, moderation in matters that have reference to behavior and our relations with the world; moderation in eating and drinking, are most excellent. But moderation in principle is a deeply-dyed absurdity. Imagine any one claiming to be a moderate mathematician or chemist, or to say that the laws of gravitation act moderately. Yet there are those who claim to be moderate homœopaths. And some even claim to practice Homœopathy or Allopathy as in their judgment may be best for their patients. Those who make such claims must be either densely ignorant or lack principle. "So then because thou art lukewarm, and neither cold nor hot, I will spew thee out of my mouth." When Galilei, Kepler, and Newton discovered nature's laws they made known what were absolute and infallible, without a shadow of variation. And when Hahnemann in the same way, and for the good of humanity, when the time was ripe and man ready to receive of the blessing of this higher law, also discovered the infallible and unfailing law of cure, his mind was intromitted into the light of which he made such blessed use:

> "As some tall cliff, that lifts its awful form,
> Swells from the vale, and midway leaves the storm,
> Though round its breast the rolling clouds are spread,
> Eternal sunshine settles on its head."

Yes, in the full knowledge of the claim we advance, we repeat that Hahnemann discovered and understood in its order, usefulness, and beauty, *similia similibus curantur*, and has left to us the way to unfailingly develop the law that has been and will continue to be a blessing to all who may be brought within the sphere of its benign influence. The wonders of the law of cure may be of use to the mind in other ways besides that of healing, grand beyond expression as this of itself is. It unfolds qualities and uses in mineral, plant, and animal never before dreamed of. Substances apparently inert, so far as medicinal uses are concerned, by division and separation of their crude particles unfold uses to our materia medica of the highest order, uses that were all unknown before Hahnemann discovered this magic wand of law. Thus are we also taught that

"Things are not what they seem."

A grain of musk will scent a room for years without any apparent diminution. How account for this? There are no known natural laws that will unfold the enigma, and we verily believe none ever will be discovered, because they are above nature and work through and by material forms, as the human mind acts through the body. There must ever and always will be some correspondential and adequate cause—*ex nihilo nihil fit.* Here Hahnemann again gives us a clue to unravel the tangled skein in the terms "dynamic," "spiritual," "vital power." If we burrow in the earth and expect to see the uses of things in external nature, we shall, like the mole, lose our sight. The sight that we deprive ourselves of is of far greater importance than that of the body; it is the perception to see dynamic, vital, and spiritual forces that are the causes of all things we see in nature. The musk is the medium through and by which something in the world of mind finds expression for man. For undoubtedly all things exist for man. Man, the microcosm, includes the gross material macrocosm with spiritual superadded. This will explain many things in nature otherwise incomprehensible. The vitally important consideration for us, as homœopathicians, is that it gives a clue to the philosophy of the law of similars, and shows its universality. It also furnishes a key to many things in Hahnemann's writings that are a stumbling-block to the mere materialist, and to the Thomases who must see, touch, and handle before they will believe.

"Herbs gladly heal our flesh,
Because they find their acquaintance there."

But we go much further than this, and in the language of Jonathan M. Sewall we assert:

> "No pent-up Utica contracts our powers,
> But the whole boundless continent is ours."

Humanity is as broad, deep, and boundless as the universe. There is no kingdom that will not minister to him: animal, vegetable, and mineral; earth, air, and sea wait his bidding for human use; all find their correlation in him; without him as their end, cause, and prototype they could not be.

Truth enlarges the mind and broadens the field of vision from whatever point it may be viewed. Thus it enables us to see the infinite diversity running through all creation, no two things ever being alike. Hence, the false doctrine of substitution has no place in the law of *similia.* Every remedy has its own peculiar individuality, and so wide is this range of action it has been found necessary to prove a remedy on different persons in order to more fully give the idea of its range of use. And in the sick we search throughout its whole range of pathogenesis for the characteristic peculiarities of the case, for, although in health a prover might not have exhibited the finer shades of differentiation, yet when disease disturbs the vital force, the patient becomes susceptible to its healing power.

Powerful poisonous doses give no true conception of these finer shades that make for use in disease until the reaction comes and the vital force begins to assert itself, and the extreme violence of the symptoms passes away. Hence, to the therapeutist strong drugs are of little service. But remedies given in potency to those who are susceptible will give the coloring, the tints, the lights, and shadows that make for use.

On this subject Dr. Hering says: "Symptoms which arise in provings of the higher potencies are similar to the later effects of the lower or so-called stronger doses."

As sickness makes for susceptibility, so may it be that those who are violently affected by a crude remedy will give the finer shades on the road to recovery; when yet in apparent health they may not be among those who would give the peculiar, characteristic, and valuable shades of differentiation. But we do not recommend any one to take crude drugs even for such a purpose. Serious injury might in some cases result. Neither do we consider it necessary. We have now some hundreds of proven remedies; and slowly, carefully, and systematically, with varying potencies on the susceptible is, we believe, the true mode of procedure.

Remedies have cured when exhibited in comparatively crude form, as well as through a wide range of potencies, from the lowest to the highest. We desire to specially emphasize our belief that the true artist need not resort to crude drugs, and that the best results can only be attained from the administration of potencies.

The late Dr. Carroll Dunham, speaking of the dose, says: "Experience must accumulate before we can discover a law for our guidance."

It seems as though in this instance the gifted doctor had not spoken with his usual clearness. Law is not the result of experience. It is God-given, not man-given. The law must be first discovered, then the more experience accumulates the more may we see the wondrous workings of God's law; the more the mind that is prepared to receive it, sees its use, beauty, and infallibility. The very fact of the learned professor stating this showed that the something he hoped would be discovered could not be a law, but only some mode to guide in the application of a law that had been already discovered. We think the doctor would have seen the force of this statement at once. Remedies have cured in a great variety of potencies, as above noted. It has been found there are cases in which some potencies act better than others. Not necessarily the highest. My own experience is that usually the potency matters little if we get the simillimum. Professor Durham gives instances in which the low acted best. I refer you to his masterly work, *Homœopathy the Science of Therapeutics.*

Mathematical accuracy can never be hoped for, either in the dose or potency. Nor do we conceive it to be in any way necessary.

Another cogent reason is that when we exhibit a potentized remedy, quantity does not enter into our thought. You cannot put into a material balance disturbances of immaterial vital forces. They are not on the same plane of being. When a pellet or pellets are given, what we see is not the remedy *per se*, any more than the material body is not the individual himself. The pellet or liquid is simply the menstruum, the vehicle by which the subtle dynamic force that permeates it, is sent on its mission. That which has been the means of segregation, which carries the remedy on its mission of love, is appropriated to functional or other uses, and carried away.

Again, you cannot weigh or measure psychological or physiological functions. For our present purpose, it suffices to refer to the latter.

Physiology can never be an exact science in the same sense as mathematics or chemistry. The conditions do not admit of it. Who can determine what the exact systole or diastole of the heart ought to be normally; or measure the number and rhythm of the respirations; or define what quantum of brain-substance is used in these varied functions? keeping them in the joy and freedom of health, yet in the golden bonds of harmony and order. They vary under all the different conditions of activity and rest, sleeping and waking, eating and drinking, with every emotion of the mind, and in hundreds of ways that will be suggested by every informed and reflecting mind.

To the true homœopath it is known that the mental and emotional symptoms are of the most importance in the selection of the remedy. Yet these cannot be put in a balance or quantitatively analyzed. How, then, can the question of bulk enter into the problem of the dose? It is relationship. As well try to measure the love you bear your child. The correlation we predicate is diverse. *Similia* is the soul of Homœopathy, and the dose the body. How shall we measure the latter? How would you have your lady love? Just as high as your heart? When the mind is absorbed with the *esse* we conceive of the *existere* as ministrant, giving tangibility, bodying forth, expressing, that it may be a medium of communication with the qualities we love and desire to be conjoined with. The painting in the true artist's mind we wot not of until ultimated on canvas. Even that only as through a glass darkly gives the author's conception; but, if understandingly we study his work we more and more appreciate and admire. But if we only see a piece of fine coloring in a gilded frame, lost to us is the spirit of the master's work; so when the mind is absorbed in externalities, their shape, size, and form, we are liable to lose sight of interior principles and qualities.

This question of dose and potency has with some been such a bone of contention that we will add another thought. Let us assume—and we have cogent grounds for the assumption—that the nerves originate, control, and regulate the functions of the organism. It follows that when the nerves are in order they obey the mandates of the will, and the whole system works in rhythm and harmony. Assuming, also, that in disease the proper mode of cure is to arouse the dormant and disorderly energies of the organism to react against malign influences, as well as against the inroads already sustained, that prevent the nerves giving out their full fruition of healthy use, it necessarily follows that the remedy to be addressed to the nerve-centres must be infinitesimal, because the nerve-cells are themselves infinitesimal. It has been estimated that there are twelve thousand to the inch, and some so small as to be incalculable. It is reasonable to suppose, therefore, that the corresponding remedy to arouse them to act throughout life's domain must even be smaller than the nerves themselves. How otherwise can they be subtle enough to penetrate their recesses and arouse them to healthy activity? Yet we have admitted that cures are sometimes effected by comparatively crude remedies. How shall we account for this? It brings us again to the consideration of the vital force. The tendency of the organism, under nervous control, is to cast off that which makes for disorder. Thus, when too much is eaten or drank, or we become too cold or too warm, or any other disturbance occurs, the excess, if not so great as to cause too much depression, is thrown off, this power of rejection varying with every one.

We opine it to be similar with the remedy: the nerve-cells, by their wondrous power, select and allow to reach their domain only what is necessary, excepting when so much has been presented as to overwhelm them in a greater or less degree. But the greatest good, the highest use, can only be attained when the remedy in its potence is in correspondence with the nerve-cells so as to be attracted along the nerve filaments without let or hindrance. In many ailments we believe that the higher potencies alone can reach the higher and finer nerve-cells. Those who have had long experience well know that the high potencies are more rapid in their action, and cure when the lower, or crude, remedies fail. We observe that some writers, while professing to be followers of Hahnemann, sometimes recommend the lancing of abscesses. We here, therefore, affirm that we have never yet found such action necessary. And for a so-called homœopathician to be considered the leading operator East, West, North, or South would throw grave doubts upon his claims to be a homœopathic purist. A writer who seems to have the faculty of providing the most liberal allowance of sack to the smallest quantum of the staff of life, has the following: "I have often felt that he is too conservative for one so skillful in operating." (*Sic.*) This sop to Cerberus was doubtless dovetailed in, in the effort to heal over the wounded surgical and other susceptibilities of a *confrère* who really needed consolation; therefore *may* be laudable from certain standards and standpoints, and should not be criticised too severely. Yet, as the attachment of the Damoclean sword that hung over the poor fellow had been so strengthened that there was little danger of its falling on his devoted head, we may be permitted to remark that it would hardly be fair to allow even so good a carver to run amuck, no matter how deft he may be, although that seems the trend of the surgery of the present day. Some such thought and practice is a necessary corollary of the surgery in vogue, otherwise the army of surgeons would find it indispensable to obtain new fields for their vocation, and this might be difficult, in view of the exceptionally lucrative character usually accruing to dexterous chirurgy. So that really the only fault to be found with the above quotation, is, that—perhaps unwittingly—it puts the matter in rather crude form, and had it appeared in a lay publication, might have caused a sense of *shock* quite natural to those outside of surgical pastures. Hence it may be seen that the cause of the present rage for surgery need not be considered of a very occult character. The *vis a tergo* of a goodly portion—or of a great deal of modern chirurgy was manifest. Not that we would condemn modern surgeons by the wholesale. We know several in whom we have the fullest confidence, and whom we would trust implicitly; we merely speak of the fashion, the rage for surgery that now unfortunately obtains. Many have the idea that cutting cures. This is a grievous mistake. Operators' measures are sometimes, though rarely, necessary, but they never cure, never eradicate a disordered condition. Medicine, and medicine alone, can do that. It should be clearly apprehended that truth admits of no middle ground; it will make no compromise. Amalek must be slain, root and branch. Because of our lack of knowledge or limitations, it may be sometimes permitted to do thus and so, as He from whom all law outflows may permit certain things because of the hardness of our hearts. In such case, however, we should admit our own shortcomings, and not throw dust into our own eyes or deceive others. The time will come when surgery will be unknown except for accidents. It is still useful for some congenital deformities, such as hare-lip and talipes, etc.; yet we believe the hereditary tendencies that produce these may be avoided by previous treatment and the study of the anamnesia in connection therewith, as has already been recorded in the literature of Homœopathy. A woman who had children with hare-lip, after a course of homœopathic treatment, gave birth to a child that did not suffer from that deformity.

And here we would, as a matter of love and duty, interpolate a few words on a subject upon which we feel most profoundly. Recently we received a communication from the American Humane Association on the subject of "Cruelty to Animals." One of the questions propounded is, "Ought it (vivisection) to be wholly abolished and treated as a crime?" This we most emphatically answer in the affirmative. We do not believe that vivisection ever gave knowledge that led to the relief of a single human being from pain, or in any way helped to ameliorate human suffering. We firmly believe that this diabolical practice is totally needless as well as dastardly inhuman. We assert that no man who has been guilty of vivisection ought to be allowed to practice as a physician. Imagine any one coming from a torture chamber to see a sick child, or to have a mission to help suffering humanity. How can one who is callous to animal suffering yearn to help his fellow-man? What can be learned from the quivering, writhing flesh for intelligent guidance in the sick? Do the tissues, laid open by the lance, display normal function? Assuredly not. What is the story that a humane mind would read? The only true one of which we can conceive is the palpitating plea for mercy, unheeded by the inhuman wretch who, in the name of a false science, gloats like a ghoul over his fiendish, bloody work. Oh! yes; but the poor brute is unconscious under an anæsthesia. And of course they are always very careful to keep him in that condition! But even were this so, is there no suffering entailed on the poor brute that silently pleads for mercy? But even if stupefied, what lesson can be learnt? Do the tissues and organs display normal function when life is outraged and laid open by the scalpel? We opine not. The only thing we can conceive of is the plea for mercy. O God! that science should be so dear and brutish torture so cheap! There is no option but to take our appeal to the court of our common humanity, even in the interest of these callous human brutes themselves, and prevent the further spread of their heartlessness on other human souls by demanding a national law making this fiendish practice a crime. We cordially indorse the lines by Cowper:

> "I would not enter on my list of friends
> (Though graced with polished manners and fine sense,
> Yet wanting sensibility) the man
> Who needlessly sets foot upon a worm."

The question arises, how ought we to regard this practice of vivisection from the standpoint of the law of *similia?* Our answer is, worse than useless. Some have attempted to prove remedies on the lower animals, but we do not see that any useful purpose has been served. The most important—*i. e.*, the subjective symptoms, it is obviously impossible to obtain. Neither can we reason from the lower to the higher—from animals to man. Knowledge on a lower plane serves little purpose for higher use, unless illumined from above. Inasmuch, however, as the greater includes the less, when conversant with the higher degrees of thought and being, our range of vision embraces all that is below. Hence, it follows we ought to be able to assist dumb creatures in their needs from the superior light the knowledge of our law vouchsafes, and so it is. Frequently have we been enabled to relieve suffering in animals from our knowledge of the materia medica. This is well known, and has been confirmed in the experience of hundreds of physicians who strive to live up to the light of God's law—*Similia similibus curantur.*

